# Effect of a *Lactobacillus* fermentation product on postweaning heifer performance

**DOI:** 10.1093/tas/txac015

**Published:** 2022-01-27

**Authors:** John B Hall, Maggie R Bloomsburg, Sandra A Goddard

**Affiliations:** 1 Nancy M. Cummings Research, Extension and Education Center, University of Idaho, Carmen, ID 83462, USA; 2 Department of Animal, Veterinary, and Food Science, University of Idaho, Moscow, ID 83844, USA

**Keywords:** efficiency, heifer, ionophore, nutrition, prebiotic, reproduction

## Abstract

The objective of the experiment was to compare the effect of dietary inclusion of a prebiotic fermentation product of *Lactobacillus acidophilus* (LaP, RumaCell; 5 mL animal^−1^ d^−1^) or monensin on performance of replacement beef heifers. Heifers received a total mixed ration containing either LaP (*n* = 77) or monensin (MON; Rumensin; 200 mg animal^−1^ d^−1^; *n* = 79). Heifers were fed for 71 d in a GrowSafe unit, so individual feed intake could be measured. Heifers were weighed every 2 wk and feed efficiency calculated by residual feed intake (RFI). At the end of the RFI trial, heifers remained on their diets for an additional 27 d and were estrus synchronized using the 14-d CIDR + PG protocol and bred by artificial insemination (AI) followed by natural service. Prior to estrous synchronization, reproductive tract scores (RTS; 1 = infantile to 5 = cycling/presence of corpus luteum) were measured. Continuous variables were analyzed using generalized mixed models, whereas categorical data were analyzed by logistic regression. Body weights, average daily gain, feed intake, and RFI value were similar (*P* > 0.30) among MON- and LaP-supplemented heifers. Across treatments, heifers gained 0.9 ± 0.1 kg/d while consuming 9.3 ± 0.5 kg of diets daily. Reproductive development as indicated by RTS was similar (*P* > 0.28) between treatments. However, estrus response increased (*P* < 0.01) and AI pregnancy rates tended to be greater (*P* < 0.07) for MON compared to LaP heifers. In contrast, the percentage of heifers pregnant by 60 and 100 d (80.4% and 90.5%, respectively) was not different (*P* > 0.33) for MON and LaP heifers. In conclusion, addition of LaP to replacement heifer diets may result in growth and reproductive performance similar to an ionophore, if dietary energy is adequate for normal heifer growth.

## INTRODUCTION

Developing replacement heifers is a critical and expensive enterprise of the cow-calf operation. Nutrition during the postweaning development phase will impact heifer reproductive development as well as direct costs (Hall, 2013). Addition of ionophores to replacement heifer diets improves average daily gain (ADG) and may decrease age at puberty (Moseley et al, 1977; Moseley et al, 1982). However, the use of ionophores is not allowed in natural and organic programs (Troxel, 2012). Ionophores may also pose a toxicity risk to monogastric livestock such as horses (Blomme et al., 1999). Therefore, the use of alternative products that produce results similar to ionophores may be advantageous.

Prebiotics and probiotics may offer an alternative to ionophores in growing cattle diets. These feed additives alter rumen microflora populations and resulting fermentation products (Dhama et al., 2008; Rai et al., 2013). However, the impact on animal productivity by these products appears to be highly variable and, at least partially, dependent on the specific product and concentration (Uyeno et al., 2015; Markowiak and Slizewska, 2018).

Previously, we reported increased ADG and dry matter intake (DMI) in steers supplemented with a *L. acidophilus* prebiotic product (LaP; RumaCell, Pacer Technologies Inc., Murtaugh, ID) compared to steers supplemented with monensin (Hall et al., 2018). The experiment was a short-term backgrounding study. The impact of LaP in developing heifers is not known, and LaP may offer an alternative to ionophores in heifer diets. Therefore, an experiment was designed to compare supplementation with LaP or monensin on prebreeding growth, DMI, feed efficiency, and pregnancy rate in confinement fed heifers. The working hypothesis was that LaP supplementation would produce results similar to monensin.

## MATERIALS AND METHODS

All procedures were approved by the University of Idaho Animal Care and Use Committee (protocol numbers 2017-61 and 2018-27).

### Animals and Experimental Design

Crossbred replacement beef heifers (*n* = 162) were stratified by age, weight, and previous preweaning and backgrounding treatments and then randomly assigned to receive diets containing either monensin (MON; 200 mg animal^−1^ d^−1^; *n* = 81) or LaP (5 mL animal^−1^ d^−1^; *n* = 81) feed additive. Heifers were born to Angus × Hereford dams and sired by Angus, Hereford, or SimAngus sires. Heifers previously had been used on projects which involved different preweaning grazing locations (range vs. pasture; Hall et al., 2020) and a backgrounding study (alfalfa vs. grass grazing; Bloomsburg, 2018). Prior nutritional management may affect heifer development (Cushman and Perry, 2019). Therefore, previous treatments were taken into consideration when allocating heifers to treatment in the present study. Heifers were weaned at an average of 207 d of age, and the backgrounding study lasted 85 d. At the beginning of the current study, heifers were similar (*P* < 0. 20) in age and weight averaging 322.1 ± 2.1 d of age and 285.5 ± 13.8 kg.

### Diets and Feeding

Heifers were fed a total mixed ration consisting of 42.5% ground alfalfa hay, 42.5% ground orchardgrass hay, 10% wheat middlings, and 5% liquid supplement on a DM basis. The molasses-based liquid supplement (PerforMix Nutrition Systems, Nampa, ID) provided minerals, vitamins, and MON or LaP (Table 1). Diets were formulated to provide 200 mg animal^−1^ d^−1^ of MON or 5 mL animal^−1^ d^−1^ of LaP. Heifers were allowed ad libitum access to diets and water.

**Table 1.** Composition of basal liquid supplement that included monensin^1^ or *L. acidophilus* prebiotic^2^

Nutrient name	Dry matter
Dry matter, %	65.50
Invert sugars, %	31.69
Crude protein, %	20.25
Crude protein as non-protein N, %	6.88
Crude fat, %	1.52
Salt, %	9.07
Calcium, %	3.15
Phosphorus, %	1.53
Magnesium, %	0.34
Potassium, %	11.15
Sulfur, %	0.52
Iron, ppm	405.50
Manganese, ppm	674.05
Zinc, ppm	840.72
Copper, ppm	269.31
Cobalt, ppm	12.21
Iodine, ppm	78.89
Selenium, ppm	5.02
Vitamin A, IU/kg	53442.67
Vitamin D, IU/kg	3817.34
Vitamin E, IU/kg	673.38
Net energy maintenance, Mcal/kg	1.65
Net energy gain, Mcal/kg	1.15
Net energy lactation, Mcal/kg	1.61

Monensin—382 mg/kg supplement.

Prebiotic fermentation product of *Lactobacillus acidophilus—*8.3 L/907 kg supplement.

The MON and LaP diets were mixed in separate feed trucks to eliminate possibility of cross contamination of diets. Feed was delivered at 0700, 1400, and 2000 h daily. Bunks were cleaned once weekly, and all orts discarded. To minimize variation in treatment, the same lots of ground hay and wheat middlings were used for both diets. However, variation in diets can occur due to small errors in the loading and mixing procedures (Vogel et al., 2015). Feed samples were collected daily from all bunks for each feed additive. Daily samples were pooled by feed additive. Daily samples were weighed and oven dried at 60 °C to determine dry matter content. Daily heifer feed intakes were adjusted for daily feed dry matter content to calculate individual animal DMI. Feed samples from each 14-d period were composited by feed additive and analyzed by near infrared spectrometry (Cumberland Valley Analytic Services, Chambersburg, PA). Nutrient content of MON- and LaP-containing diets was compared to ensure that there were no differences other than the MON or LaP supplement (Table 2).

**Table 2.** Comparison of nutrient analysis of diets^1^ supplemented with monensin^2^ or LAP^3^ fed to developing replacement beef heifers during a 98-d trial

Component	Feed additive		Std Err	*P*-Value
	Monensin	LaP		
Dry matter (DM),%	92.3	92.7	0.18	0.22
Crude protein, %DM	9.3	9.8	0.34	0.36
Acid detergent fiber, %DM	46.7	45.8	0.52	0.27
Neutral detergent fiber, %DM	64.8	63.1	0.99	0.25
Ash, %DM	7.38	7.96	0.15	0.03
Ca, %DM	0.48	0.55	0.03	0.16
P, %DM	0.19	0.21	0.01	0.01
Mg, %DM	0.16	0.17	0.01	0.28
K, %DM	2.18	2.25	0.07	0.42
Total digestible nutrients, %	53.5	53.0	0.85	0.66
Net energy maintenance, Mcal/kg	0.50	0.49	0.02	0.62
Net energy gain, Mcal/kg	0.24	0.23	0.01	0.69

Basal diet was a total mixed ration consisting of 42.5% ground alfalfa hay, 42.5% ground orchardgrass hay, 10% wheat middlings, and 5% liquid supplement on a DM basis. Liquid supplement contained the feed additives.

Monensin—200 mg animal^−1^ d^−1^.

3LaP (RumaCell; prebiotic fermentation product of *Lactobacillus acidophilus*), 5 mL animal^−1^ d^−1^.

All heifers were fed in a GrowSafe System (GrowSafe Systems Ltd, Model 6000 Calgary, AB) consisting of 5 nodes per pen and 2 pens per MON and LaP treatment for a total of 4 pens. Each pen was designed to provide enough physical and GrowSafe bunk space to support 40 to 45 heifers per pen. A 14-d warm-up period was followed by a 98-d experimental period. After the 14-d warm-up period, 6 heifers were removed from the experiment due to failure to eat out of the GrowSafe bunks resulting in 77 MON and 79 LaP heifers, respectfully.

### Data Collection

Heifers were weighed on two consecutive days at the beginning of the experiment (days 1 and 2), at the end of the residual feed intake trial (days 70 and 71), and end of the experiment (days 97 and 98). Consecutive weights were averaged. In addition, heifers were weighed every 2 wk during the experiment. Beginning and final weights were used to calculate trial ADG. Individual animal feed intakes were recorded daily. Diet dry matter was determined daily for each diet and used to calculate individual animal daily DMI. Fat thickness between the 12th and 13th ribs was measured by ultrasound at the conclusion of the residual feed intake (RFI) trial. Residual feed intake was calculated based on methodology previously used in our laboratory (McGee et al., 2013). The final formula was RFI = DMI − [1.75 + 2.09 (ADG) + 0.081 (metabolic mid-point BW) − 0.046(rib fat thickness)]. Heifers were assigned to RFI groups as Efficient (<−0.5 S.D.), Average (−0.5 S.D. < X < 0.5 S.D.), or Inefficient (> 0.5 S.D.).

After the conclusion of the RFI feeding period, heifers remained in their respective pens and were fed the same diet until the conclusion of artificial insemination. Prior to estrous synchronization, heifers were weighed, body condition scored, and reproductive tracts scored. Body condition scores were from 1 = emaciated to 9 = obese (Herd and Sprott, 1986). Reproductive tracts scores (RTS) were from 1 = Infantile to 5 = Cycling/corpus luteum present (Anderson et al, 1991). After prebreeding evaluations, eight heifers (*n* = 3 MON; *n* = 5 LaP) were eliminated from breeding due to size or an RTS = 1. Heifers were estrus synchronized using the 14-d CIDR + PG protocol (Mallory et al., 2010). Briefly, heifers received a controlled internal drug release (CIDR; 1.38 g of progestin; Zoetis) device for 14 d followed by an injection of PGF_2α_ (25 mg i.m.; Lutalyse; Zoetis) 16 d after CIDR removal and artificially inseminated (AI) with one of two Angus bulls at 72 h after PGF_2α_ administration. An estrus detection aid (Estrotect; Estrotect Innovative, Spring Valley, WI) was applied to each heifer at the time of PGF_2α_ administration. Fourteen days after AI, clean-up bulls were introduced for an additional 30 d. Pregnancy status was determined via ultrasonography at 60 d and via palpation at 100 d after AI. Ultrasound examination at 60 d was used to differentiate AI sired fetuses from natural service sired fetuses.

### Statistics

Animal was the experimental unit as the GrowSafe system allows for calculation of individual animal intakes. The data analysis for this paper was generated using SAS software (v9.4), Copyright © 2016 SAS Institute Inc., Cary, NC. Body weights were analyzed by generalized mixed model with repeated measures using MIXED procedures. The model included feed additive, time and feed additive × time interaction as fixed effects with feed additive × backgrounding regime, sire breed, summer grazing location, and feed additive × backgrounding regime × time as random effects. Generalized mixed model was used to analyze each weighing event ADG, trial ADG, and trial weight gain included fixed effect of feed additive, backgrounding regime, and feed additive × backgrounding regime interaction with pen × feed additive, sire breed, and summer grazing location as random effects.

Residual feed intake and DMI were analyzed by generalized mixed model with fixed effect of feed additive, backgrounding regime, and feed additive × backgrounding regime interaction with pen, sire breed, and summer grazing location as random effects. General mixed model (GLIMMIX) analysis using a multinomial categorical model was used to compare percentage of heifers LaP and MON heifers in each RFI group. Logistic regression modeling the probability of pregnancy at AI, 60 d, and 100 d was used. Models were adjusted for body condition, BW, and age.

For all analyses, a 95% confidence interval was used with P < 0.05 considered significant and P < 0.10 considered a tendency.

## RESULTS

Across all analyses, there were no effects (*P* > 0.50) of preweaning grazing location or backgrounding regime or their interactions with feed additive. However, these fixed effects remained in the model.

**Figure 1.**
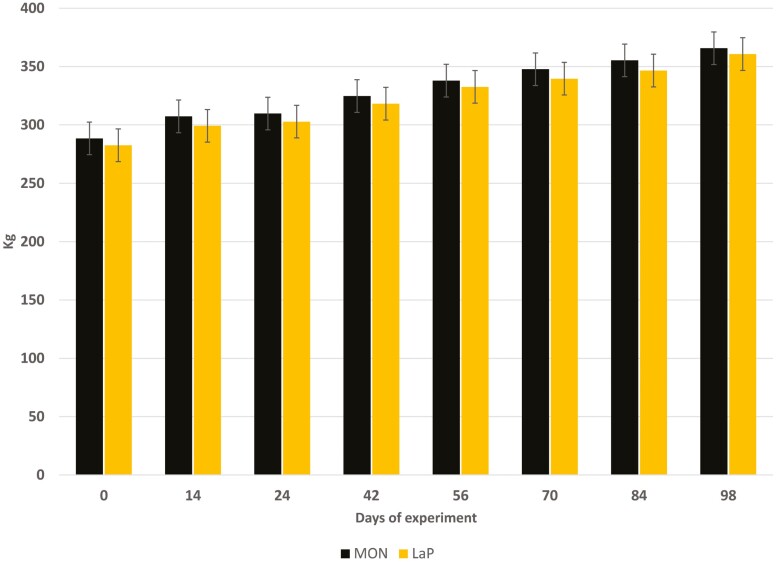
Changes in body weight of replacement beef heifers receiving a total mixed ration (85% hay, 15% concentrate) containing monensin (MON; Rumensin; 200 mg animal^−1^ d^−1^; *n* = 79; black bars) or a prebiotic fermentation product of *Lactobacillus acidophilus* (LaP, RumaCell; 5 mL animal^−1^ d^−1^; *n* = 77; gold bars). Diets were fed for 98 d. No effect of feed additive on body weight was detected (*P* > 0.17). Day effect (*P* < 0.05).

Body weights were similar (*P* = 0.169) for heifers receiving MON or LaP at all weighing events. Body weights increased (*P* < 0.0001) over time (Figure 1) and there was no feed additive × time interaction (*P* = 0.99). Overall experiment ADG was similar (*P* < 0.99) between MON and LaP heifers averaging 0.9 ± 0.1 kg/d. Average daily gain by weighing event was not affected (*P* > 0.26) by feed additive; however, gains among weighing events varied (*P* < 0.05; Table 3).

**Table 3.** Average daily gains (kg/d ± SE) for replacement beef heifers receiving monensin (MON) or prebiotic fermentation product of *Lactobacillus acidophilus* (LaP) containing liquid supplements in a total mixed ration during a 98-d trial^1^

Days of experiment	Feed additive		*P*-value
	MON	LaP	
0 to 14	1.5 ± 0.1^a^	1.3 ± 0.1^a^	0.26
14 to 28	0.3 ± 0.2^b^	0.5 ± 0.2^b^	0.31
28 to 42	1.0 ± 0.2^c^	1.1 ± 0.2^c^	0.82
42 to 56	1.0 ± 0.2^c^	1.0 ± 0.2^c^	0.90
56 to 70	0.9 ± 0.6^b,c^	0.6 ± 0.6^b,c^	0.79
70 to 84	0.6 ± 0.4^b,c^	0.5 ± 0.4^b,c^	0.90
84 to 98	0.7 ± 0.2^b,c^	0.9 ± 0.2^c^	0.27

Basal diet was a total mixed ration consisting of 42.5% ground alfalfa hay, 42.5% ground orchardgrass hay, 10% wheat middlings, and 5% liquid supplement on a DM basis. Liquid supplement contained the feed additives. Monensin—200 mg animal^−1^ d^−1^; LaP = RumaCell*—*5 mL animal^−1^ d^−1^.

Within columns, means with different superscripts differ (*P* < 0.05).

Dry matter intake was similar (*P* = 0.83) between heifers receiving MON and LaP averaging 9.2 ± 0.5 and 9.4 ± 0.5 kg/d, respectively. Average RFI did not differ (*P* = 0.45) between feed additives (−0.106 ± 0.30 and 0.059 ± 0.30, for MON and LaP, respectively). Percentage of heifers in RFI groups was not affected (*P* = 0.54) by feed additive. Distribution by RFI group for MON and LaP, respectively, was Efficient (28.6%, 31.6%), Average (45.5%, 34.2%), and Inefficient (26.0, 34.2%).

Prebreeding BW and RTS were not different (*P* > 0.28) between MON and LaP heifers (Table 4). Prebreeding body condition score was greater (*P* < 0.01) for LaP compared to MON heifers (Table 4). The percentage of heifers expressing estrus after synchronization was increased (*P* < 0.01) in MON compared to LaP. There was a tendency for more MON heifers to conceive to AI than LaP heifers (*P* < 0.07). However, pregnancy rates were similar at 60 d (*P* = 0.33) and 100 d (*P* = 0.66) after AI for heifers consuming MON or LaP, and across feed additives averaged 80.4% and 90.5% for 60 and 100 d, respectively.

**Table 4.** Body weights, body condition, and reproductive responses for replacement beef heifers receiving monensin (MON; *n* = 76) or prebiotic fermentation product of *Lactobacillus acidophilus* (LaP; *n* = 72) containing liquid supplements in a total mixed ration during a 98-d trial^1^

Item	Feed additive		*P*-value
	MON	LaP	
Body weight (kg)	359.4 ± 3.8	353.4 ± 3.8	0.26
Body condition score	5.8 ± 0.1	6.2 ± 0.1	0.02
Reproductive tract score	3.3 ± 0.1	3.1 ± 0.1	0.22
Estrus response (%)	77.0 ± 5.0	56.5 ± 6.1	0.01
AI pregnancy rate (%)	58.0 ± 5.8	42.4 ± 5.9	0.07
60-d Pregnancy rate (%)	83.6 ± 4.4	77.2 ± 5.1	0.33
100-d Pregnancy rate (%)	89.4 ± 3.6	91.6 ± 3.3	0.66

Basal diet was a total mixed ration consisting of 42.5% ground alfalfa hay, 42.5% ground orchardgrass hay, 10% wheat middlings, and 5% liquid supplement on a DM basis. Liquid supplement contained the feed additives.

Monensin—200 mg animal^−1^ d^−1^; LaP = RumaCell*—*5 mL animal^−1^ d^−1^.

## DISCUSSION

Enhancing growth rate or improving feed efficiency may reduce the costs associated with replacement beef heifer production. In general, heifers are developed to reach 55% to 65% of their mature weight by breeding (Hall, 2013). Rate of gain to achieve this developmental goal usually varies from 0.45 to 0.80 kg/d from weaning until breeding (Patterson et al., 1992; Hall, 2013). Optimum rate of gain is dependent on weaning weight and percent of mature weight desired. Since the post-weaning development period can be 8 to 9 mo long, feed costs become a significant factor. Feed additives that improve weight gain or feed efficiency are one strategy to reduce heifer development costs.

This study compared growth, feed intake, and reproductive responses of peripuberal heifers to a feed additive which improves growth and feed efficiency, monensin, to heifers receiving the prebiotic, LaP. Overall, the prebiotic-treated heifers responded similarly to the ionophore-supplemented heifers. Previous experiments with prebiotics in cattle focused on supplementation of pre-ruminant calves with oligosaccharides (Quigley et al., 2002; Heinrichs et al., 2003, Ghosh and Mehla, 2012; Grand et al., 2013). In general, these experiments demonstrated beneficial effects on health and growth through alterations in the hindgut microbiome. Most studies involving cattle with functional rumens examined the effects of probiotics, in contrast to prebiotics, on animal performance and rumen function (see reviews by Uyeno et al., 2015; Retta, 2016). More recently, studies compared the effects of probiotics or synbiotics (pre- and probiotic combination) to effects of monensin on growth and/or health in growing/finishing cattle (Columbo et al., 2021; Neves et al., 2021). The current experiment is among the few reports of the impacts of this *L. acidophilus* prebiotic on cattle performance.

It is well established that ionophores, such as monensin, improve growth rate and feed efficiency in cattle (Potter et al., 1976; Raun et al., 1976; Goodrich et al., 1984). In growing replacement heifers, inclusion of monensin in the diet increased growth rate and decreased age at puberty compared to diets without monensin (Moseley et al, 1977; Moseley et al, 1982). Since the positive effects of monensin when included in a total mixed ration, with sufficient energy, are well documented in the literature, we designed the study to use MON as the reference or control diet.

Actual average intake of feed additives based on DMI and inclusion rate in the total mixed ration was 177.6 mg animal^−1^ d^−1^ and 4.26 mL animal^−1^ d^−1^ for MON and LaP, respectively. Therefore, average feed additive intake was ≥ 85% of target for both treatments. Positive effects of monensin in a range of doses from 50 to 400 mg animal^−1^ d^−1^ are well documented with 200 mg animal^−1^ d^−1^ considered an effective dose for growth promotion (Goodrich et al., 1984; Potter et al., 1976; Kunkle et al., 2000). Monensin increases ADG and feed efficiency in supplemented animals compared to unsupplemented controls (Goodrich et al., 1984; Kunkle et al., 2000). Therefore, we are confident that the performance of the MON heifers reflects the positive effect of monensin, but a negative control was not part of the experimental design. There are no available published dose–response studies for LaP; however, the inclusion rate in this study was according to the recommendations of the manufacturer.

Dry matter intake for both groups was only 0.25 to 0.30 kg per day greater than that predicted by the Nutrient Requirements of Beef Cattle (NRBC, National Academies 2020). Similarly, BW and growth rates were not markedly different between treatments. Animal performance, as indicated by ADG, agreed with NRBC projections for yearling cattle receiving an ionophore. Previously, we observed an increase in DMI and ADG in steers receiving LaP compared to steers receiving MON (Hall et al., 2018). In that study, animals received a higher concentrate, lower roughage diet (25% concentrate; Hall et al., 2018) than animals in the present study (15% concentrate). In addition, the treatment period was only half the duration of the present study. Whether the previously observed impacts of LaP on DMI and ADG are dependent on dietary energy content or duration of treatment is not clear and warrants further study.

The growth response was similar among LaP- and MON-treated heifers. As previously mentioned, addition of monensin improves growth rate and feed efficiency. With high energy, high concentrate feedlot diets, animals receiving monensin had increased feed efficiency, which was a result of both increased ADG and reduced DMI (Goodrich et al., 1984). In contrast, when monensin was included in diets of animals grazing pasture or consuming high roughage diets, feed intake was not suppressed, but modest improvements in ADG (0.05 to 0.21 kg/d) resulted in minor improvements in feed efficiency (Potter et al., 1976; Moseley et al., 1982; Kunkle et al., 2000). In high roughage diets, increases in growth rate in response to monensin may be dependent on ad libitum access to feed (Moseley et al., 1982). In the present study, animal access to feed was not restricted. In agreement with the present study, Colombo et al. (2021) observed no difference in growth rates in feeder cattle consuming receiving diets that included a synbiotic or a monensin-tylosin feed additive. Adding a prebiotic to finishing diets containing monensin did not alter growth rate in steers (Pancini et al., 2020). In contrast, diets including a probiotic increased ADG in steers compared to diets containing monensin (Neves et al., 2021).

The lack of difference between DMI in MON and LaP in the present study would be consistent previously described limited effects of monesin on DMI in high forage diets (Potter et al., 1976; Kunkle et al., 2000). Colombo et al. (2021) observed a transient increase in DMI in steers receiving diets containing a synbiotic, but this increase was not maintained and trial DMI was similar among treatments. Probiotics either as an alternative to monensin or an additive to monesin containing diets did not improve DMI (Pancini et al., 2020; Neves et al., 2021). The design and animal numbers used in the present study, admittedly, may allow for potential type II statistical error. As growth and intake responses were similar between MON- and LaP-supplemented heifers, we cautiously conclude that LaP inclusion in replacement beef heifer diets results in similar performance as addition of an ionophore. As there are few studies examining the effect of LaP on animal performance, additional studies are warranted.

Consistent with the lack of effect of treatment on growth or dry matter intake, feed efficiency, as determined by average RFI value, was not altered in the present study. Similarly, distribution of heifers in efficient, average, or inefficient groups was not impacted by treatment. Residual feed intake estimates feed efficiency regardless of productivity, and it measures the deviation from predicted intake for a particular level of performance (Herd and Arthur, 2009). Approximately 19% of the variation in RFI can be attributed to digestive and heat increment alterations. Alterations in rumen microbial populations have been associated with differences in RFI (Elolimy et al., 2018). Although we had previously described alterations in fermentation due to LaP (Hall et al., 2018), LaP did not result in changes in efficiency in the current study.

Positive reproductive responses may be related to increased growth rate and greater propionate production in monensin-supplemented heifers (Mosley et al., 1977; Mosley et al., 1982; Lalman et al., 1993). Reported differences in pregnancy rate between control and monesin supplemented heifers range from 0% to 19% in favor of monensin (Mosley et al., 1977; Mosley et al., 1982; Lalman et al., 1993). Often the number of animals in these studies limits the ability to detect differences below 20%. The information on *L. acidophilus* supplementation on reproduction in large ruminants is limited. Work from El-Nagar et al. (2021) demonstrated improved reproductive performance in lactating Egyptian buffaloes in response to oral supplementation with a *L. acidophilus* probiotic.

In the present study, MON and LaP heifers had a similar rate of reproductive development as indicated by RTS. However, MON heifers had improved estrous response to estrous synchronization and a tendency for increased pregnancy rates to artificial insemination compared to LaP heifers. Monensin supplementation increases responsiveness of the ovary to gonadotrophins compared to controls (Bushmich et al., 1980). An increase in ovarian responsiveness to estrous synchronization may explain the improvements in early pregnancy rates in the present study. However, by 60 and 100 d after initiation of the breeding season, those differences were no longer apparent. In this study, the detection limit for differences in reproductive traits was 15% due to the number of animals per treatment. Therefore, only dramatic differences in reproductive traits could be detected. Large-scale reproductive experiments on the effects of pre- and probiotics are needed.

Animal response to prebiotics or probiotics can be highly variable and may be dependent on a variety of factors including health or age of the animal, percentage of concentrates in the diet, or type of prebiotic/probiotic provided (Dhama et al., 2008; Rai et al., 2013; Uyeno et al., 2015). Uyeno et al. (2015) reviewed studies examining the effects of probiotics (*n* = 8) or prebiotics (*n* = 8) on performance and health in calves. Although 71% of the probiotic trials examined resulted in increased weight gain compared to controls, only 25% of the prebiotic studies demonstrated a growth advantage. Only 25% of both prebiotic and probiotic studies reported increases in feed efficiency, whereas 50% of trials indicated health benefits.

Prebiotics are usually fermentable ingredients, which may alter bacterial populations in the large intestine or rumen through stimulation or suppression of specific species (Rai et al., 2013; Markowiak and Slizewska, 2018). Most commercially available prebiotics for livestock are poly- or oligosacchraides. Response to prebiotics in non-ruminants include increases in *Lactobacillus* and *Bifidobacterium* with concomitant reductions in *Salmonella* and *E. coli* (Markowiak and Slizewska, 2018). In general, these microbial responses also translated into enhanced animal health and performance. However, response to prebiotics may depend on species as prebiotics benefits were noted in pigs and chickens but not turkeys (Markowiak and Slizewska, 2018). As previously noted, most of the studies reviewed on prebiotics in calves resulted in little or no response (Uyeno et al., 2015). Response to prebiotics may be affected by diet as well. Calves supplemented with cellooligosaccaride and fed whole milk exhibited positive responses, whereas calves fed milk replacer did not respond to the prebiotic (Uneyo et al., 2015). Grains that differed in carbohydrate composition resulted in alterations in the intestinal microbiome of piglets, and these dietary-induced alterations may also affect response to the prebiotic (Markowiak and Slizewska, 2018).

The prebiotic used in the present study is a *L. acidophilus* fermentation product, which contains spent *Lactobacillus* cells, organic compounds of fermentation, and the media on which it was grown (RumaCell, Pacer Technologies). Based on this information, it is not clear what components of this product may have the greatest effect on animal response or the precise mechanism of action. Previously, we reported reductions in production of propionate and increases in butyrate, valerate, and isovalerate in vitro between LaP compared to MON supplementation (Hall et al., 2018). *Lactobacillus acidophilus* produces the bacterioncins lactocin B, lactacin F, acidocin A, and acidocin B (Gopal, 2011). Whether these bactericidal compounds are still present in the product or have activity in the rumen are not known.

In summary, the use of a *L. acidophilus* fermentation product as a prebiotic may be a viable substitute for ionophores in replacement heifer diets. Based on the conditions of the present study, growth and reproductive responses to this type of prebiotic appear to be similar to monensin in diets containing sufficient energy to support recommended growth rates in heifers. Further investigation on *L. acidophilus* fermentation products on growth as well as focusing on dose-response, rumen function, and mechanisms of action is warranted.
